# Effects of gliadin and glutenin on the hygroscopicity of freeze-dried apple powders

**DOI:** 10.3389/fnut.2022.894176

**Published:** 2022-09-28

**Authors:** Xiaotong Yang, Yujie Wei, Jing Liu, Hongshan Liang, Bin Li, Jing Li

**Affiliations:** ^1^College of Food Science and Technology, Huazhong Agricultural University, Wuhan, China; ^2^Wuhan Sunma Biotechnology Co., Ltd., Wuhan, China

**Keywords:** apple powder, anticaking agent, water migration, wheat gluten, gliadin, glutenin, moisture adsorption

## Abstract

Wheat gluten addition in freeze-dried apple powders can effectively prevent their undesirable moisture adsorption and caking during long-term storage, but the working mechanism of wheat gluten had not been expounded. Therefore, such anti-hygroscopicity effects were systematically investigated from the perspective of wheat gluten major components: gliadin and glutenin. Herein, moisture adsorption curve/isotherm, morphology, and moisture migration law of the protein-added apple powders were analyzed at varied storage humidities. Results showed that Peleg, GAB, and Ferro-Fontan models could describe the moisture adsorption process of gliadin-added and glutenin-added freeze-dried apple powder. By comparing the model fitting results, it was found that the fitting degree of moisture adsorption isotherm of the sample increased with the increase of water activity, and the imitative effect of the Ferro-Fontan model was the best. According to the result of the fitting prediction, the equilibrium moisture content of glutenin-added apple powder was 4.7% lower than that of gliadin-added apple powder at 25°C and 75% relative humidity (RH). Type III moisture adsorption isotherms were observed for gliadin-added apple powder, while that of glutenin-added apple powder was type II. In addition, the gliadin-added apple powder demonstrated better fluidity and lower water migration when the relative humidity (RH) of the environment was lower than 58%. Once above this RH value, the protecting effect of glutenin was more obvious. These findings not only elucidate the anti-hygroscopic mechanism of wheat gluten in the processing of apple powder, but also provide a new idea for improving the quality of apple powder and the development of new anti-hygroscopic agents.

## Introduction

The overcapacity in apple production has necessitated the development of its value-added end products. Apple powder is a new food ingredient that can be used in most areas of food processing and more importantly can transform the overproduced apple into more convenient food. Physicochemical properties such as moisture content, hygroscopicity, solubility, fluidity, color, and particle size are important indexes when evaluating fruit/vegetable powders (1). However, apple powder is particularly susceptible to moisture and temperature variations during long-term storage, which leads to undesirable moisture adsorption, caking, and deterioration of powder sensory performance (2). To reduce the hygroscopicity of food powders and maintain its fluidity, appropriate use of anticaking agent is inevitable (3). Farahnaky et al. added maltodextrin into jujube powder, and the modified powder exhibited increased glass transition temperature and reduced adsorption moisture capacity (4). Jakubczyk et al. found that adding maltodextrin could significantly reduce the moisture adsorption of apple powder (5). Fang et al. found that adding 1% protein to bayberry powder could significantly increase the extraction rate of spray drying, and adding 30% maltodextrin could achieve the same rate of powder extraction (6). Wang et al. added cherry pomace to corn starch, which significantly reduced the moisture adsorption of corn starch (7). Chang et al. added tricalcium phosphate and calcium silicate into soursop powders, which inhibited its moisture adsorption and recovery rate (8). Amrutha et al. found that the oligofructose solution with 2% magnesium oxide had the highest powder yield and good fluidity after spray drying (9). Addo et al. reported that calcium stearate, magnesium stearate, and silica could obviously restrain the moisture adsorption and caking of jujube powder (10). In addition, many types of anticaking agents are also used in the food industry, such as β-cyclodextrin, whey protein isolate (WPI), soluble starch, and pectin (11–13).

In recent years, the pursuit of natural food additives has accelerated the development of high-performance anti-hygroscopic agents from edible resources, such as proteins, polysaccharides, and other bio-macromolecules. Wheat gluten is an abundant plant protein from the byproduct of wheat, corn, and other cereals. According to our preliminary research, wheat gluten exhibited a strong inhibiting effect of moisture adsorption of freeze-dried apple powder. Moreover, gliadin and glutenin are the major components in wheat gluten, showing different physiochemical properties (14). In this study, the moisture adsorption curve/isotherm, macroscopic morphology, and migration law of internal moisture of gliadin-added apple powder and glutenin-added apple powder were, respectively, characterized. The difference of the inhibition effect of two kinds of proteins on the moisture adsorption behavior of apple powder system was explored. In this study, natural plant protein was used as anti-hygroscopic agents in the processing of apple powder, which not only improved the quality and safety of products, but also provided scientific basis and validations for the development of new anti-hygroscopic agents in the food industry.

## Materials and methods

### Materials

Apple was bought at local supermarket in Wuhan, China. Wheat gluten was bought from Huafeng Powder Industry Co., Ltd. (Henan, China). Hydrochloric acid, sodium hydroxide, dichloromethane, absolute ethyl alcohol, lithium chloride, potassium acetate, potassium carbonate, sodium bromide, potassium iodide, and sodium chloride were chemical reagents and purchased from Sinopharm Chemical Reagent Co., Ltd. (Shanghai, China).

### Preparation of samples

#### Preparation of gliadin

According to the method of Hong et al. and van den Broeck et al. (15, 16), the wheat gluten and dichloromethane are mixed according to a ratio of 1:10 (w/v), and an electric stirrer (HD2010W, Sile Instrument Co., Ltd., Shanghai, China) was used for degreasing at 400 r/min for 3 h. Then remove the dichloromethane by suction filtration and dry for 12 h. Repeat this step twice to obtain the defatted wheat gluten. Defatted wheat gluten flour was dissolved in 70% ethanol at a ratio of 1:10 (w/v) and stirred with an electric stirrer at 400 r/min for 3 h. After standing for 12 h, a high-speed centrifuge (H1850R, Xiangyi Dynamic Test Instrument Co., Ltd., Hunan, China) was used to centrifuge it at 9,000 g at 4°C for 10 min. The supernatant fluid was evaporated at 40°C, and the obtained precipitate was freeze-dried at vacuum degree < 1 Pa for 48 h in a freeze dryer (LGJ-30FD, Songyuan Huaxing Technology Development Co., Ltd., Beijing, China) and collected as the gliadin, which was mechanically crushed for before use.

#### Preparation of glutenin

Defatted wheat gluten was dissolved in distilled water at a ratio of 1:15 (w/v), and the pH value was adjusted to 10. After centrifugation, the supernatant fluid was added with anhydrous ethanol to prepare 65% ethanol solution, and the pH was adjusted to 7.0. After standing for 12 h at 4°C, centrifugation was performed. The precipitate was washed with distilled water three times and freeze-dried under vacuum degree <1 pa to obtain the glutenin, which was also mechanically crushed (17).

#### Preparation of freeze-dried apple powder

The mature and undamaged apples were washed, peeled, and cut into cubes, and the apple pulp was obtained by crushing for 60 s with a mechanical crusher (model MB-1001, Homax, 30,000 r/min). Then, the apple pulp (AP) was mixed with distilled water in a 100-ml beaker, followed by the addition of extracted gliadin (Gli) or glutenin (Glu) powders at the mass ratios (AP/Gli otr AP/Glu) of 7:3, 1:1, 3:7, and 0:10. After mechanically stirring (HD2010W, Sile Instrument Co., Ltd.) at 600 rpm for 30 min, eight samples were prefrozen at −20°C for 24 h and then freeze-dried for 48 h with vacuum degree < 1 Pa (LGJ-30FD, Songyuan Huaxing Technology Development Co., Ltd., Beijing, China). The obtained samples were mechanically crushed. Then, the samples were sieved, and the particle size was between 100 mesh and 120 mesh. Accurately weighed sample powders (1 g) were placed in a weighing bottle with constant weight (25 × 40 mm), and its mass was recorded. The weighing bottle containing samples was placed in a vacuum desiccator containing P_2_O_5_ to balance moisture. Its mass was recorded until constant weight (± 0.0003 g). Then, the sample powder was sealed for further testing.

### Determination of moisture adsorption curve

Apple powders were accurately weighted (marked as M_0_) and placed in a constant weight weighing bottle (25 × 40 mm, the quality was marked as M_1_), which was then opened and placed in a sealed desiccator containing different saturated salt solutions at constant temperature (25°C). Specifically, the environment relative humidity of the desiccator containing LiCl, CH_3_COOK, MgCl_2_, K_2_CO_3_, NaBr, KI, and NaCl saturated solution was 11.30, 22.51, 32.78, 43.16, 57.57, 68.86, and 75.29%; that is, the corresponding water activity was 0.11, 0.22, 0.33, 0.43, 0.58, 0.69, and 0.75. The weight of the sample-loaded weighing bottle (M_2_) was measured at 0, 3, 6, 12, and 24 h, and then, it was measured every 24 h until 240 h (2). All measurements were replicated three times, and the average value was used to draw the moisture adsorption curve. The moisture adsorption content (X) of the sample was calculated as follows:


(1) $$\text{X}=\left({\text{M}}_{\text{2}}-{\text{M}}_{\text{1}}-{\text{M}}_{0}\right)/{\text{M}}_{0}$$

where M_0_ was the weight of the initial sample, M_1_ was the weight of the bottle, and M_2_ was the weight of the bottle containing the hygroscopic samples.

### Determination of moisture adsorption isotherms

In the same way as in 2.3, the sample powder was placed in the sealed desiccator with different relative humidity and kept at the constant temperature (25°C). It was measured every 24 h until change in weight loss or gain reached less than 0.0003 g for two successive readings. At this point, the sample reached hygroscopic equilibrium state, the mass of the sample powder (in triplicate) was recorded, and the equilibrium moisture content (EMC) was calculated by Equation (1) (18). Then, GAB, Ferro-Fontan, Smith, Henderson, Oswin, and Peleg models were selected for fitting. The coefficient of determination (R^2^), residual sum of squares (RSS), and root mean square error (RMSE) were used as goodness-of-fit statistics, and the moisture adsorption isotherm of the sample was drawn by combining the model fitting curve with the highest fitting degree.

### Macroscopic analysis of freeze-dried apple powder

When reaching the moisture adsorption equilibrium, the apple powder was placed in a black background and photographed with a digital camera to observe its macroscopic morphology, dispersion, and the degree of adhesion and caking.

### Measurement of low-field nuclear magnetic resonance

A nuclear magnetic resonance analyzer (NMI20-015V-I, Niumag Co., Ltd., Suzhou, China) was used to detect the transverse relaxation time (T_2_) of freeze-dried powders of AP/Gli and AP/Glu (0:10, 1:1, and 7:3 in mass ratio) that reached the moisture adsorption balance. Samples were weighted (0.2 ± 0.01 g) and wrapped in PTFE to prevent evaporation of water during the test and put into the nuclear magnetic tube for detection. Carr–Purcell–Meiboom–Gill sequence (CPMG) scanning experiment was conducted for analysis. The parameters of detection settings were as follows: the detection temperature was 32°C; the proton resonance frequency (SF) was 21 MHz; the spectral width was 100 kHz; the repeat interval time (TW) was 3,000 ms; the number of repeated scans (NS) was 16; the relaxation decay time (TE) was 0.16 ms; the number of echoes was 4,000. Multi-Exp Inv Analysis software was used to invert the T_2_ decay curve and obtain the T_2_ relaxation time spectrum of the sample (19).

### Data analysis

The models were fitted by Origin 9. The R^2^, RSS, and RMSE were calculated by Origin 9 and used as goodness-of-fit statistics. In addition, the data points in the figure were all the average values of triplicate measurements. The data were expressed as the mean ± S.D. (standard deviation), and *p* < 0.05 was considered statistically significant. The statistical analyses were performed by SPSS software (SPSS version 25.0, IBM Institute, USA).

## Results and discussion

### Moisture adsorption curve

**FIGURE 1:**
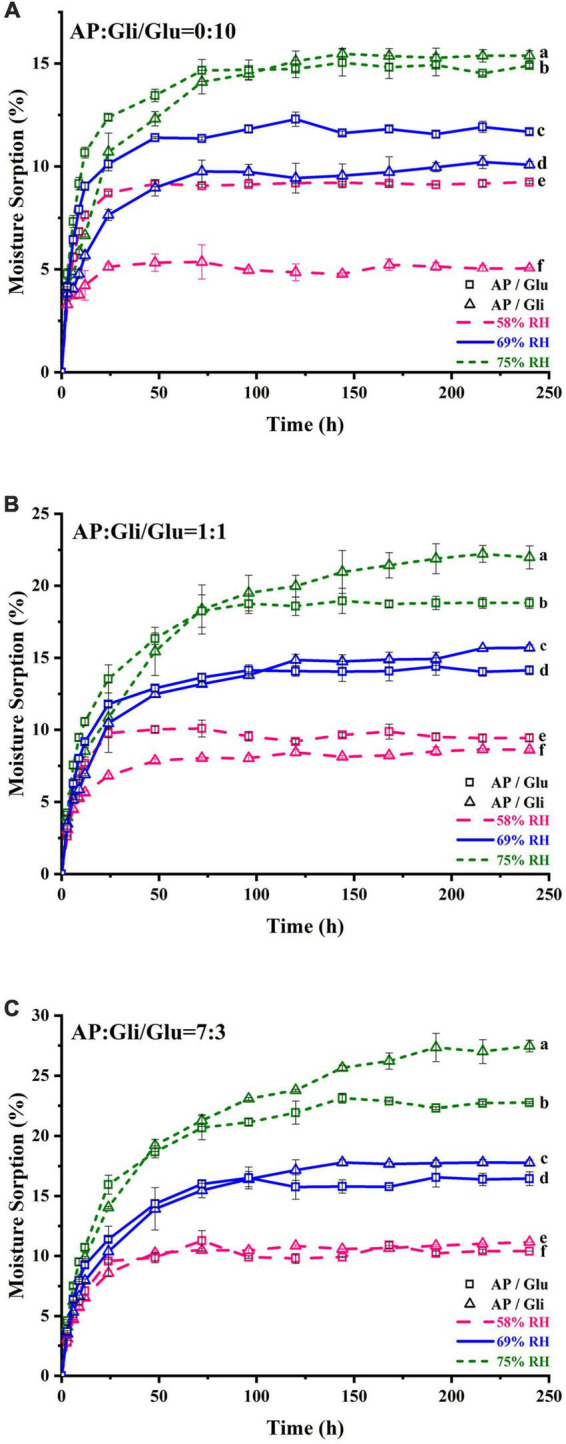
Moisture adsorption curve of samples with the ratio of AP and Gli or Glu at 0:10 **(A)**, 1:1 **(B)**, and 7:3 **(C)**. The data points were the average values of triplicate measurements. Different lowercase letters indicated that the equilibrium moisture content of different samples was significantly different (*p* < 0.05).

Figure 1 shows the moisture adsorption behaviors of apple pulp (AP) and two kinds of freeze-dried apple powder with protein mass ratios of 0:10, 1:1, and 7:3 at the relative humidity (RH) of 58, 69, and 75%. The EMC of pure gliadin was 5.04% at the RH of 58%, which was significantly (*p* < 0.05) lower than that of pure glutenin (9.24%) (Figure 1A). When the RH rose to 69%, the two proteins exhibited similar moisture adsorption curves. At the RH of 75%, the moisture adsorption of gliadin was lower than that of glutenin before 103 h. After that, the EMC of gliadin was significantly (*p* < 0.05) higher than that of glutenin. Those results indicated that the hygroscopicity of the two proteins was quite different under moderate RH, while they are more or less the same at medium and high humidity.

When the apple pulp and protein were mixed at mass ratio of 1:1, the moisture adsorption content of powders with gliadin was lower than those added with glutenin at the RH of 58%. As the storage time increased, their moisture adsorption content became the same when the RH rose to 69%. At the RH of 75%, the moisture adsorption of the sample added with gliadin before 75 h was lower than that of the sample added with glutenin. After 75 h, the moisture adsorption of the sample added with glutenin was lower, and it was 3.1% lower when it reached the moisture adsorption equilibrium, which was 28 h earlier than the intersection point in Figures 1A,B). When the mass ratio of apple pulp to protein was 7:3, the EMC of the sample with two kinds of protein added was almost the same at the RH of 58%. Then, the curve became crossed at the RH of 69%. At the RH of 75%, the intersection point was 30 h earlier than that in Figure 1B, and the EMC difference of the samples with two kinds of protein was increased to 4.7% (Figure 1C).

In conclusion, for pristine protein samples, the moisture adsorption of gliadin was lower than that of glutenin in an environment of less than the RH of 69%, while the moisture adsorption of gliadin was higher than that of glutenin in an environment of more than the RH of 69%. When the mass ratio of AP to two kinds of protein was 7:3, the inhibition effect of gliadin on hygroscopicity was better when the relative humidity of the storage environment was lower than 58%. If the relative humidity of the storage environment was higher than 58%, the inhibition effect of glutenin on hygroscopicity of the system was better. In addition, when the moisture adsorption of freeze-dried apple powder with the same amount of two kinds of protein was less than 15%, the moisture adsorption of the sample added with gliadin was lower.

### Moisture adsorption isotherms

#### Model fitting of moisture adsorption isotherms

Seven classical models were used to process the moisture adsorption test data stored at 25°C in seven different relative humidity environments. The fitting correlation coefficients (R^2^) are shown in Table 1 and Table 2. The results showed that the fitting effect of the moisture adsorption isotherm model of gliadin-added sample was Peleg > GAB > Ferro-Fontan > Oswin > Hendenson > Smith. The fitting effect of the moisture adsorption isotherm model of glutenin-added sample was Peleg > GAB > Ferro-Fontan > Oswin > Hendenson > Smith. For the samples containing gliadin and glutenin, the fitting correlation coefficients of Peleg, GAB, and Ferro-Fontan were all above 0.99, which indicated that these three models were more suitable for fitting the moisture adsorption isotherms of the sample.

**TABLE 1:** Statistical coefficients of mathematical models of AP/Gli samples with different proportion.

Model	AP/Gli = 0:10			AP/Gli = 1:1			AP/Gli = 7:3		
			
	R^2^	RSS	RMSE	R^2^	RSS	RMSE	R^2^	RSS	RMSE
GAB	0.996	0.801	0.316	0.998	0.678	0.291	0.998	1.186	0.385
Ferro-Fontan	0.994	1.010	0.355	0.999	0.425	0.230	0.998	1.531	0.437
Smith	0.900	18.309	1.513	0.932	30.344	1.948	0.932	45.327	2.380
Henderson	0.945	10.010	1.119	0.995	2.029	0.504	0.992	5.273	0.812
Oswin	0.965	6.351	0.891	0.998	0.833	0.323	0.997	2.111	0.514
Peleg	0.999	0.185	0.152	1.000	0.067	0.091	0.998	1.423	0.422

**TABLE 2:** Statistical coefficients of mathematical models of AP/Glu samples with different proportion.

Model	AP/Glu = 0:10			AP/Glu = 1:1			AP/Glu = 7:3		
			
	R^2^	RSS	RMSE	R^2^	RSS	RMSE	R^2^	RSS	RMSE
GAB	0.998	0.761	0.309	1.000	0.123	0.124	0.999	0.224	0.167
Ferro-Fontan	0.993	1.248	0.395	0.999	0.278	0.187	1.000	0.099	0.111
Smith	0.993	1.254	0.396	0.981	5.435	0.824	0.964	15.885	1.409
Henderson	0.995	0.938	0.343	0.983	4.822	0.776	0.991	4.187	0.723
Oswin	0.995	1.013	0.356	0.994	1.663	0.456	0.998	1.047	0.362
Peleg	1.000	0.071	0.094	0.999	0.144	0.134	0.999	0.283	0.188

#### Model fitting test of freeze-dried apple powder

The EMC measured values of freeze-dried apple powder with the mass ratios of apple pulp and two kinds of proteins of 1:1 and 7:3 were compared with the predicted values of Peleg, GAB, and Ferro-Fontan models. As shown in Tables 3–6, the average relative errors of Peleg, GAB, and Ferro-Fontan models for apple powder samples were 2.38–17.51, 1.41–13.15, and 2.23%–10.07%, respectively. In addition, the fitting results of each model showed a similar trend. At medium-to-high water activity (a_*w*_), the relative error between the measured value and predicted value of all freeze-dried apple powder samples was small and the fitting degree was high, while the relative error was large at low a_*w*_. It indicated that these models were more effective in predicting the EMC of freeze-dried apple powder with medium-to-high a_*w*_. Ferro-Fontan was the best model to fit the moisture adsorption isotherms of freeze-dried apple powder. It had important guiding significance for predicting the EMC of freeze-dried apple powder, as well as for drying, storage, and packaging of freeze-dried apple powder.

**TABLE 3:** Predicted values compared with measured values of EMC of AP/Gli = 1:1 apple powder.

a_*w*_	Measured value	Peleg		GAB		Ferro-Fontan	
				
		Predicted value	Relative error/%	Predicted value	Relative error/%	Predicted value	Relative error/%
0.75	0.2198	0.2194	0.1820	0.2204	0.2730	0.2208	0.4550
0.69	0.1570	0.1578	0.5096	0.1551	1.2102	0.1547	1.4650
0.58	0.0862	0.0854	0.9281	0.0873	1.2761	0.0865	0.3480
0.43	0.0387	0.0389	0.5168	0.0418	8.0103	0.0420	8.5271
0.33	0.0243	0.0255	4.9383	0.0252	3.7037	0.0261	7.4074
0.22	0.0196	0.0173	11.7347	0.0133	32.1429	0.0141	28.0612
0.11	0.0099	0.0106	7.0707	0.0054	45.4545	0.0075	24.2424

**TABLE 4:** Predicted values compared with measured values of EMC of AP/Gli = 7:3 apple powder.

a_*w*_	Measured value	Peleg		GAB		Ferro-Fontan	
				
		Predicted value	Relative error/%	Predicted value	Relative error/%	Predicted value	Relative error/%
0.75	0.2746	0.2744	0.0728	0.2733	0.4734	0.2724	0.8012
0.69	0.1776	0.1688	4.9550	0.1828	2.9279	0.1849	4.1104
0.58	0.1116	0.1079	3.3154	0.1036	7.1685	0.1027	7.9749
0.43	0.0506	0.0571	12.8458	0.0538	6.3241	0.0521	2.9644
0.33	0.0312	0.0331	6.0897	0.0347	11.2179	0.0341	9.2949
0.22	0.0211	0.0144	31.7536	0.0198	6.1611	0.0209	0.9479
0.11	0.0096	0.0035	63.5417	0.0087	9.3750	0.0116	20.8333

**TABLE 5:** Predicted values compared with measured values of EMC of AP/Glu = 1:1 apple powder.

a_*w*_	Measured value	Peleg		GAB		Ferro-Fontan	
				
		Predicted value	Relative error/%	Predicted value	Relative error/%	Predicted value	Relative error/%
0.75	0.1882	0.1879	0.1594	0.1879	0.1594	0.1876	0.3188
0.69	0.1414	0.1419	0.3536	0.1416	0.1414	0.1424	0.7072
0.58	0.0945	0.0944	0.1058	0.0958	1.3757	0.0960	1.5873
0.43	0.0663	0.0640	3.4691	0.0633	4.5249	0.0623	6.0331
0.33	0.0478	0.0506	5.8577	0.0492	2.9289	0.0479	0.2092
0.22	0.0360	0.0363	0.8333	0.0361	0.2778	0.0357	0.8333
0.11	0.0222	0.0209	5.8559	0.0223	0.4505	0.0251	13.0631

**TABLE 6:** Predicted values compared with measured values of EMC of AP/Glu = 7:3 apple powder.

a_*w*_	Measured value	Peleg		GAB		Ferro-Fontan	
				
		Predicted value	Relative error/%	Predicted value	Relative error/%	Predicted value	Relative error/%
0.75	0.2275	0.2262	0.5714	0.2278	0.1319	0.2269	0.2637
0.69	0.1638	0.1591	2.8694	0.1635	0.1832	0.1655	1.0379
0.58	0.1041	0.1012	2.7858	0.1026	1.4409	0.1024	1.6330
0.43	0.0589	0.0587	0.3396	0.0607	3.0560	0.0588	0.1698
0.33	0.0403	0.0428	6.2035	0.043	6.6998	0.0416	3.2258
0.22	0.0290	0.0287	1.0345	0.0274	5.5172	0.0279	3.7931
0.11	0.0163	0.0151	7.3620	0.0137	15.9509	0.0172	5.5215

**FIGURE 2:**
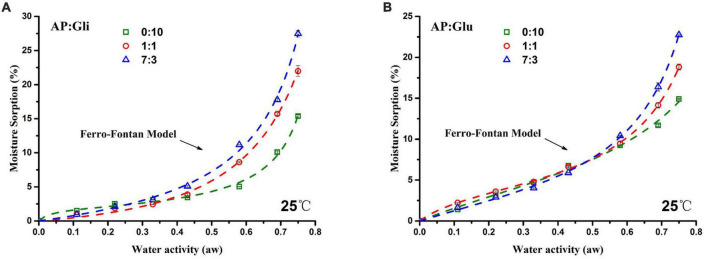
Moisture adsorption isotherms of samples with mass ratios of AP/Gli **(A)** and AP/Glu **(B)** of 0:10, 7:3, and 1:1.

The results of equilibrium moisture adsorption of gliadin-added and glutenin-added freeze-dried apple powders were fitted by the Ferro-Fontan model (in the range of 0.11 a_*w*_ to 0.75 a_*w*_), and the obtained moisture adsorption isotherm is shown in Figure 2. The moisture adsorption isotherm moved down with increased protein content. The equilibrium moisture content of freeze-dried apple powder increased slowly in the low a_*w*_ period and increased rapidly when a_*w*_ exceeded 0.58, which was a typical pattern of high sugar food (4).

According to Brunauer classification (20), by calculating the slope value of the moisture adsorption isotherm, it could be judged that both moisture adsorption isotherms of gliadin and glutenin were typical “S” shape, which belonged to type II moisture adsorption isotherms. The samples with AP/Gli mass ratios of 1:1 and 7:3 showed type III moisture adsorption isotherm (Figure 2A). However, the samples with AP/Glu mass ratios of 1:1 and 7:3 showed the same type II moisture adsorption isotherm as that of pure protein (Figure 2B). In addition, in the low a_*w*_ range, the EMC of glutenin-added apple powder was higher than that of the gliadin-added apple powder. In the high a_*w*_ range, the EMC of the gliadin-added apple powder was higher than that of the glutenin-added apple powder. Therefore, when the moisture adsorption content of the sample was less than 15%, gliadin played a major role in the anti-hygroscopic effect of freeze-dried apple powder, while glutenin played a major role when the moisture adsorption content was above 15%.

### Macroscopic morphology

**FIGURE 3:**
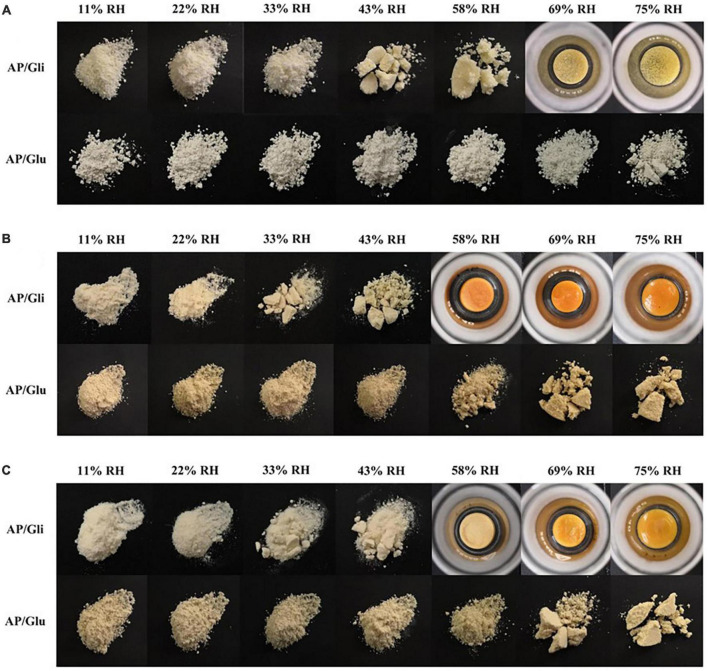
Macroscopic photos of the morphology of the samples with mass ratio of AP/Gli and AP/Glu of 0:10 **(A)**, 7:3 **(B)**, and 1:1 **(C)** at different relative humidity.

Macroscopic observations of gliadin and glutenin at their moisture adsorption equilibrium under seven different relative humidity environments are shown in Figure 3A. Agglomerate occurred for glutenin at the RH of 43%. When the RH attained 69%, the proteins shrank into a chunk owing to their excessive moisture adsorption. However, glutenin was loose under various relative humidity conditions, and there was still only a slight agglomeration even at the RH of 75%. In consistent with the results in Section 3.1, the moisture content of glutenin was higher than that of gliadin at the RH of 58% and 69%. At the RH of 75%, glutenin was more effective in maintaining the state of powder particles. At the mass ratio of AP/Gli and AP/Glu of 7:3, the gliadin-containing powders began to agglomerate at RH higher than 33% (Figure 3B). At increased RH (58%), the sample powder became contracted and chunked, and the color also deepened gradually. In terms of glutenin-added powders, no caking was observed even at the RH of 58%. At the mass ratio of AP/Gli and AP/Glu of 1:1 (Figure 3C), the caking of freeze-dried apple powder added with gliadin could be crushed by slight external force at the RH of 33% and 43%. The sample added with gliadin formed a hard agglomerate when the RH reached 58%. The freeze-dried apple powder added with glutenin did not agglomerate until the RH reached 69%. All these results indicated that glutenin had a significant inhibitory effect on the caking of freeze-dried apple powder during storage at high relative humidity compared with gliadin. This might be due to the superior power fluidity of gluten after absorbing moisture.

### Dynamic migration of moisture

**FIGURE 4:**
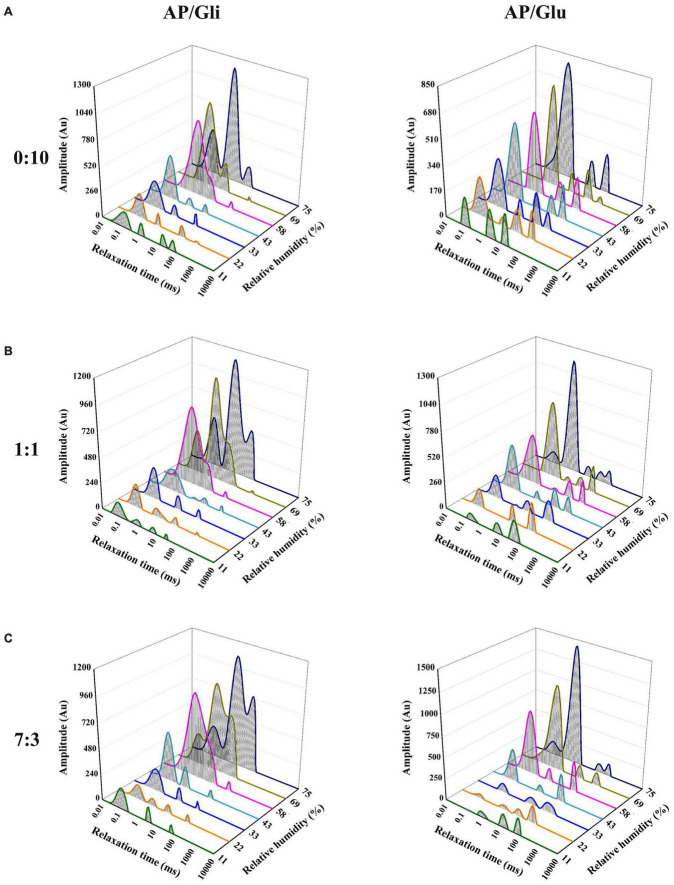
T_2_ relaxation time distribution curves of the samples with mass ratio of AP/Gli and AP/Glu of 0:10 **(A)**, 1:1 **(B)**, and 7:3 **(C)** at different relative humidity.

The T_2_ relaxation time distribution curves of the samples made from apple pulp mixed with gliadin and glutenin are shown in Figure 4, which could be used to compare the differences of the effects of the two proteins on the water state in the system. The relaxation time of bound water (T_21_), stagnated water (T_22_), and free water (T_23_) was 0.01 ms–1 ms, 1 ms–20 ms, and 10 ms–1000 ms, respectively.

As shown in Figure 4A, the state of water molecules in glutenin was relatively stable as RH increased. The change mainly showed the gradual increase of bound water content, and the peak time gradually moved to the right from 0.11 ms to 0.66 ms. However, gliadin began to appear double humps at the RH of 58%, which indicated the dynamic migration of bound water to stagnant water. Figure 4C shows the T_2_ relaxation time distribution curves of freeze-dried apple powder with the mass ratio of apple pulp to protein of 7:3. Gliadin-added powder began to show water migration at the RH of 58%. However, the water content of freeze-dried apple powder added with glutenin was transformed into stagnant water at the RH of 75%. At the mass ratio of apple pulp to protein of 1:1, higher proportion of gliadin could effectively reduce the amount of water migration in the freeze-dried apple powder sample to higher degree of freedom, while higher proportion of glutenin had little effect on the water migration (Figure 4B).

**FIGURE 5:**
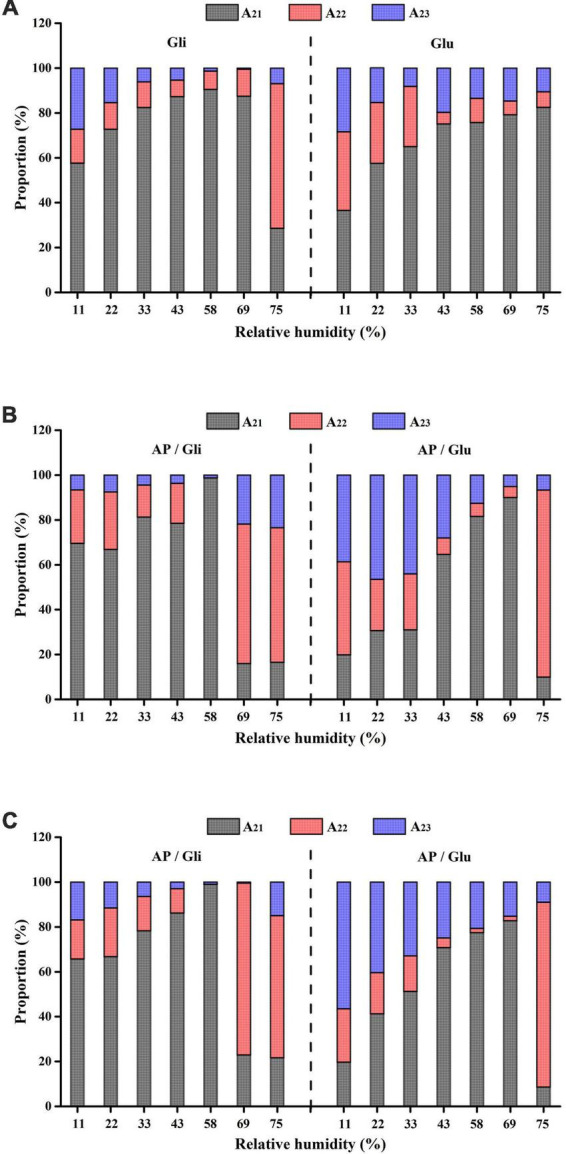
Variation of peak area ratio of various moisture in freeze-dried apple powder with mass ratio of AP/Gli and AP/Glu of 0:10 **(A)**, 7:3 **(B)**, and 1:1 **(C)** at different relative humidity.

To better display the dynamic migration law of water and the conversion of water types in the hygroscopic process of different samples, the peak areas of corresponding signals of bound water, stagnated water, and free water (A_21_, A_22_, and A_23_) were compared. As shown in Figure 5A, when RH increased from 11% to 58%, the proportion of stagnated water and free water in gliadin gradually decreased, while the proportion of bound water kept increasing, up to 90.51%. It demonstrated that the absorbed water constantly migrated into the state of bound water. When RH was greater than 69%, the proportion of hysteretic water increased, indicating that the water began to migrate to the stagnated water. However, the proportion of bound water in glutenin was always the highest, which might be the reason for the agglomeration of glutenin under different RH conditions. Figure 5B shows that the migration of bound water to stagnated water occurred in the freeze-dried apple flour with AP/Gli and AP/Glu of 7:3. The difference was that the migration of gliadin-added freeze-dried apple powder occurred earlier and the system contained more free water, which resulted in a greater degree of agglomeration of the powder. As shown in Figure 5C, compared with the first two kinds of freeze-dried apple powder, the proportion of bound water in the freeze-dried apple powder with the mass ratio of AP to Gli 1:1 increased, while the proportion of free water decreased under higher RH. However, the distribution of water types in freeze-dried apple powder with the mass ratio of AP to Glu 1:1 had no obvious change. The three types of water converted to each other with the increase of ambient RH. The reason for this may be that at the initial stage of hygroscopic behavior, capillary action within and between powder particles had the ability to absorb water, and water adhered to the surface of particles or filled the gap. As more water was absorbed, water gradually penetrated and bonded with proteins and sugars by hydrogen bonds. When the hydrophilic groups of glucose residues in freeze-dried apple powder absorbed water and expanded, more hydrophilic binding sites might be exposed, and the water molecules would migrate to a more free state, resulting in adhesion between particles.

To sum up, the water in the gliadin-added sample migrated to the direction of high degree of freedom earlier. Before the water migration in the system, the proportion of bound water in the gliadin-added sample was significantly higher than that of the glutenin-added sample, which could maintain the powder quality better.

## Conclusion

The moisture adsorption curve and moisture adsorption isotherm showed that gliadin played a major role in the anti-hygroscopic effect of gluten on freeze-dried apple powder when the moisture adsorption content was lower than 15%, while glutenin played a major role when the moisture adsorption content was higher than 15%. From the macroscopic morphology of the samples, glutenin obviously restrained the caking effect of freeze-dried apple powder during storage at high relative humidity. T_2_ relaxation time distribution curves showed that water redistribution in freeze-dried apple powder was more restricted by glutenin than gliadin. To sum up, glutenin played a major role in changing the moisture adsorption behavior of freeze-dried apple powder, while the gliadin-containing powders exhibited better powder fluidity in low humidity environment. Our study elaborated on the anti-hygroscopicity effect of gluten for freeze-dried apple powder and shed new light for the development of high-quality apple powder ingredients.

## Data availability statement

The raw data supporting the conclusions of this article will be made available by the authors, without undue reservation.

## Author contributions

JLi and BL: conceptualization. JLi: supervision and funding acquisition and critical revision of the manuscript. JLi, YW, XY, and JLiu: methodology. JLi, XY, and YW: investigation and data curation. XY, YW, JLiu, and HL: formal analysis. XY and JLi: writing—original draft. JLi, XY, and BL: writing—review and editing. All authors contributed to the article and approved the submitted version.
